# The Role of the Left Atrium: From Multimodality Imaging to Clinical Practice: A Review

**DOI:** 10.3390/life12081191

**Published:** 2022-08-04

**Authors:** Matteo Beltrami, Lorenzo-Lupo Dei, Massimo Milli

**Affiliations:** 1 Cardiology Unit, San Giovanni di Dio Hospital, 50142 Florence, Italy; 2 Cardiology Unit, Department of Life, Health and Environmental Sciences, University of L’Aquila, 67100 L’Aquila, Italy

**Keywords:** left atrial size, left atrial function, heart failure, atrial fibrillation, ischaemic cardiomyopathy, hypertension, mitral insufficiency

## Abstract

In recent years, new interest is growing in the left atrium (LA). LA functional analysis and measurement have an essential role in cardiac function evaluation. Left atrial size and function are key elements during the noninvasive analysis of diastolic function in several heart diseases. The LA represents a “neuroendocrine organ” with high sensitivity to the nervous, endocrine, and immune systems. New insights highlight the importance of left atrial structural, contractile, and/or electrophysiological changes, introducing the concept of “atrial cardiomyopathy”, which is closely linked to underlying heart disease, arrhythmias, and conditions such as aging. The diagnostic algorithm for atrial cardiomyopathy should follow a stepwise approach, combining risk factors, clinical characteristics, and imaging. Constant advances in imaging techniques offer superb opportunities for a comprehensive evaluation of LA function, underlying specific mechanisms, and patterns of progression. In this literature review, we aim to suggest a practical, stepwise algorithm with integrative multimodality imaging and a clinical approach for LA geometry and functional analysis. This integrates diastolic flow analysis with LA remodelling by the application of traditional and new diagnostic imaging techniques in several clinical settings such as heart failure (HF), atrial fibrillation (AF), coronary artery disease (CAD), and mitral regurgitation (MR).

## 1. Introduction

### 1.1. Multimodality Imaging to Improve Patient Care

In recent years, heart associations such as the European Association of Cardiovascular Imaging (EACVI) put much effort into establishing a Task Force on Multimodality Imaging (TFMMI) to implement multimodality imaging and improve patient care. A considerable amount of research has been directed towards establishing the best working conditions, such as the geographical and administrative colocation of services and staff, to bring together imaging expertise for shared reporting and decision making. Many challenges need to be overcome to develop a multimodality imaging approach, such as managerial, financial, personal, and regulatory barriers. Collaboration between cardiologists and nuclear medicine and radiology specialised physicians is key to offering the most clinically effective and cost-effective testing for the patient. Additional effort should be made to obtain competence in additional imaging modalities out of personal single-modality expertise.

The strategy scientific associations can implement to reach the best possible outcome for patient care is to find enhanced solutions through education and research of the multimodality approach [1].

### 1.2. Left Atrial Anatomy

The LA is the most posterior chamber of the heart, separated from the oesophagus by the fibrous pericardium. The anatomy of the LA consists of four components: (1) a portion that receives blood from pulmonary veins (PVs); (2) a vestibule, which surrounds the mitral orifice; (3) the interatrial septum; and (4) the left atrium appendage (LAA). All these components, apart from the LAA, do not have clear anatomic demarcations.

The pulmonary veins (PVs) enter from the posterior wall, enclosing a prominent atrial dome. The walls of the LA can be divided into superior, posterior, left lateral, septal, anterior, and posteroinferior walls. Its anterior wall is immediately behind the aortic root [2]. The oesophagus is located behind the LA posterior wall, while the left phrenic nerve runs close to the left PVs down the left side of the heart. The relative position of the left PVs to these two structures may change with LA dilatation.

### 1.3. Neuroendocrine Activity and Left Atrial Function

**Figure 1:**
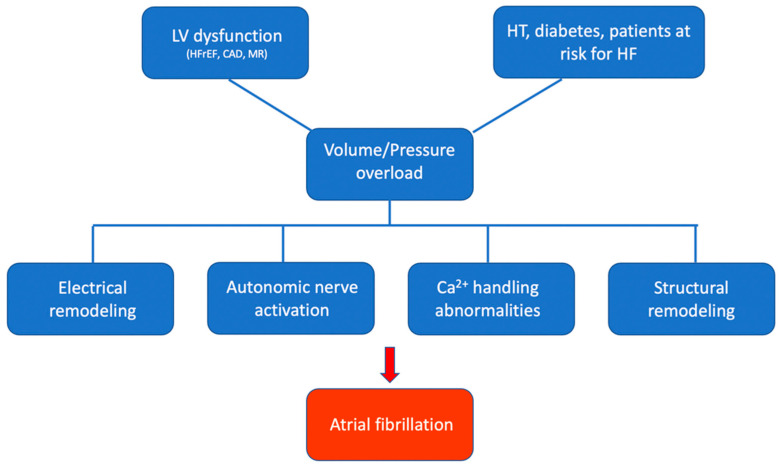
Electrical remodelling, autonomic nerve activation, and calcium handling abnormalities facilitate the generation of ectopic firing, providing triggers for re-entry initiation. The vulnerable substrate for such re-entry is represented by HT, diabetes, and areas of ischaemia, inflammation, and fibrosis in the atria (structural remodelling). CAD: coronary artery disease; HFrEF: heart failure with reduced ejection fraction; HF: heart failure; HT: hypertension; LV: left ventricular; MR: mitral regurgitation.

The left atrial activity consists of three phases. The “reservoir” phase, in which the LA receives the blood from pulmonary veins; then there is a “conduit” phase, during the first part of the ventricular diastole, which lets blood flow into the left ventricle (LV); finally, in the last phase of the diastole, the atrial contraction phase occurs. This latter moment of the atrial cycle is called the “booster pump”. If the diastolic function is not impaired, the three phases account for about 40%, 35%, and 25% of LV filling, respectively [3]. This whole atrial cycle is influenced by the capacity of the LA to relax, as well as by its stiffness and contractility. The latest LA phase becomes even more important in guaranteeing LV filling in patients with LV dysfunction, as the missing of the “booster pump” phase during AF significantly affects the hemodynamic cycle of the LV [4]. In healthy individuals, LA function is usually normal until the sixth decade, after which a functional decline begins, leading to diastolic dysfunction [5]. The LA should be considered a “multifunctional and neuroendocrine organ” with high sensitivity to the nervous, endocrine, and immune systems. The LA contributes to endocrine secretion in response to specific similar stimuli such as hypertension, diabetes, myocardial ischaemia, and valvular disease [6]. Volume and pressure overload are the main stimuli for LA endocrine activation. The difficulty in LV filling depends on diastolic dysfunction, impaired LV compliance, elevated end-diastolic ventricular filling pressure, LA and LV pressure gradients, and impaired LV contractile function. Having a stiffer LV translates into an increased end-diastolic pressure, especially during effort, and a decreased stroke volume during atrial contraction [7]. These changes are due to LA ionic channels, cellular energy properties, neurohormonal expression, and inflammatory response. In patients with HF, there is an increase in the plasmatic concentration of atrial and brain natriuretic peptides secondary to the activation of the sympathetic and renin–angiotensin–aldosterone systems [5]. The chronic activation of neurohormonal activity leads to a profibrotic, proarrhythmic, and prothrombotic state associated with LA remodelling [8]. The sympathetic activity and the consequent chronotropic stimulation increase LA and pulmonary pressure. The acute response to LA wall stretch is responsible for atrial natriuretic peptide secretion. Atrial myocytes secret natriuretic hormones in response to fluid homeostasis and blood pressure. Inflammation, LA fibrosis, and alterations in calcium channels change the homeostasis of the LA and initiate the process that leads to AF (Figure 1). The inappropriate ventricular response, atrial overload, and LA dysfunction activate the neurohormonal system. AF stimulates the secretion of atrial natriuretic peptide (ANP), and the evaluation of anatomical and endocrine activity after electrical cardioversion remains debated. The restoration of LA function after one month of electrical cardioversion could be partial, and these results explain the high level of ANP also in patients that return to sinus rhythm [9]. On the other hand, ANP and brain natriuretic peptide (BNP) levels are reduced in patients with paroxysmal atrial fibrillation (PAF), without structural heart disease, after successful radiofrequency catheter ablation [10].

## 2. Combining Multimodality Imaging Improves the LA Assessment

**Figure 2:**
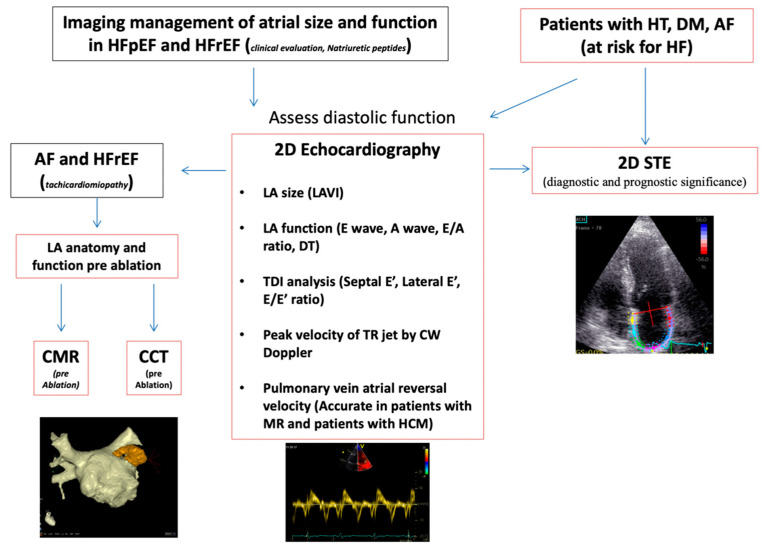
Proposed algorithm to assess left atrial size and function in the management of HFpEF and HFrEF and in patients with systemic disease at risk of HF. AF: atrial fibrillation; BMI: body mass index; CCT: cardiac computed tomography; CMR: cardiac magnetic resonance; DM: diabetes mellitus; DT: deceleration time velocity; E/Vp: the ratio between mitral E to colour M-mode flow propagation velocity; HCM: hypertrophic cardiomyopathy; HF: heart failure; HFpEF: heart failure with preserved ejection fraction; HFrEF: heart failure with reduced ejection fraction; HT: hypertension; IVRT: isovolumic relaxation time; LA: left atrial; LAVI: left atrial volume index; MR: mitral regurgitation; STE: speckle tracking echocardiography; TDI: tissue Doppler imaging; TR: tricuspidal regurgitation.

Multimodality imaging is useful to evaluate the LA anatomy, size, and function (Figure 2). Currently, echocardiography is the most cost-effective technique to assess LA size and function [11]. Currently, the left atrial volume index (LAVI) is the gold standard to measure LA size. Instead, to measure LA volume, the most validated tools are biplane Simpson’s method and biplane area length by four and two-chamber views [12]. Moreover, the evaluation of LA function can be useful to evaluate LV diastolic function. This can be achieved by evaluating LA stroke volume and LA ejection fraction [13]. Recent interesting results showed the clinical relevance of adding LA strain to the LAVI to detect LV diastolic dysfunction (LVDD) in patients with preserved LV ejection fraction. Unfortunately, the 2D echocardiography LAVI significantly underestimates LA volume when compared with cardiac magnetic resonance (CMR) or cardiac computed tomography (CCT), especially in an enlarged LA [14,15]. In comparison, 3D echocardiography displays a better agreement with CMR imaging [16]. LAVI-3D echocardiography has superior clinical value compared with LAVI-2D echocardiography, since it analyses LA volumes in the different phases of the atrial cycle, also adding automated border detection. LA stroke volume (LAVmax–LAVmin) and LA ejection fraction (LA stroke volume/maximum LA volume × 100) are assessed by 3D echocardiography. These are strongly associated with LV diastolic function parameters. Recently, 3D echocardiography reference values for LA volumes and function have been provided [17].

CMR is the gold standard to evaluate heart chambers’ volumes and functions due to its high spatial resolution. The LA is assessed in the short axis, which is more consistent than the biplane area length [18]. Late gadolinium enhancement (LGE) evaluation and feature tracking are the most important tools to evaluate LA anatomy, function [19], and the extent of LA fibrosis [20].

The assessment of LA cardiomyopathy is key to achieving a better understanding of left atrial disease progression. Chronic electrical, mechanical, and metabolic stressors remodel atrial structure. These processes involve the myocyte and extracellular matrix, the altered expression of cellular ionic properties, and autonomic nervous system abnormalities [21]. The subsequent inflammation process creates fibrotic scars and electrical remodelling that impair global atrial function. In this setting, low voltage areas (LVAs), a surrogate of LA fibrosis, could be the target to assess the left atrial remodelling and its clinical consequence. However, the relationship between LVAs, LA volume, and fibrosis is complex; left atrial scarring can be detected by CMR and significantly correlates with reduced electrogram amplitudes, as recorded by endocardial voltage maps. LVAs correlate with atrial fibrosis and with N-Terminal pro atrial natriuretic peptide (NT-proANP) plasma levels at the beginning of the atrial remodelling process. Surprisingly, LVAs do not consistently correlate with atrial volume, since there could be patients with intensely fibrotic LA but with normal volume. In a chronic setting, the presence of LVAs does not correlate with NT-proANP, because the more the LA cardiomyopathy progresses, the more the fibrotic tissue, and the less LA myocardium there is to secret ANP [22].

CCT has recently emerged as a strong tool to assess LA and pulmonary veins’ anatomy [23]. The CCT study of LA can estimate the risk of AF recurrence and worse outcomes in patients with atrial dilatation and rearrangement [24]. Images of the LA, collected by CCT, are also useful to identify the risk of CV disease (such as heart failure (HF), AF, and stroke) progression in the general population.

## 3. Left Atrial Function in Heart Failure

### 3.1. Left Atrium in Heart Failure with Preserved Ejection Fraction

Heart failure with preserved ejection fraction (HFpEF) is an increasingly prevalent clinical condition with different pathophysiological mechanisms and phenotypes. LA dilatation and impaired function are both recognised markers of hemodynamic severity. They represent an expression of the pathophysiological mechanisms of HFpEF and correlate with a worse quality of life [25]. The E/E’ ratio has an essential role in the echocardiographic diagnosis of HFpEF, indirectly estimating LV filling pressure [26,27]. However, LA stiffness is the strongest tool to identify subjects with HFpEF [28]. Even if LV diastolic function is preserved at rest, the LAVI can predict severity status. An increased LAVI expresses the severity and duration of augmented LA pressure caused by LV diastolic dysfunction. It also predicts mortality in patients with HFpEF [29]. Impaired LA strain and larger volume during effort are both related to impaired exercise tolerance. When HFpEF and RV dysfunction coexist, LA compliance is the best independent predictor of limited exertion tolerance [30]. In an acute HF setting, patients with HFpEF show an average left atrial pressure (LAp) > 24 mmHg, while stable patients show such values only in a percentage < 5%. These patients with Lap > 24 mmHg, in the chronic setting, have higher rates of hospitalisation for HF. LA work is assessed by the echocardiographic measure of LA kinetic energy (LAKE) by the following formula: 0.5 × m × A (2), where m is LA stroke volume × blood density, and A is transmitral Doppler peak atrial velocity. The more the LV becomes hypertrophic and/or impaired in diastolic function, the more the LA work increases to guarantee LV filling. LAKE is five times higher in patients with chronic HF (CHF) than in healthy controls, and it is strongly related to cardiovascular (CV) events and hospitalisation for HF [31]. In every HF setting, LA size and work are mainly increased by diastolic dysfunction. Santos et al. observed impairment in the three phases of LA function during HF. Based on their findings, the systolic LA strain is decreased independently of LA size or history of AF, showing that LA dysfunction could be a marker of HF severity [32]. LA size and increased diastolic myocardial stiffness are strictly related. The atrial diastolic wall strain index could be a useful tool to evaluate impaired diastolic function; patients with more advanced diastolic stiffness show progressive impairment of diastolic filling, with worse long-term outcomes in patients with HFpEF [33]. LA performance analysis by using CMR feature tracking evaluates LA longitudinal strain and identifies patients with LV diastolic dysfunction [34]. LA conduit function impairment is a characteristic of HFpEF, independently of LV stiffness and diastolic function. The conduit function is not deeply linked to LV diastolic function, hinting that the LA conduit function is representative of intrinsic LA pathology. Moreover, there is a high correlation between LA conduit strain and peak oxygen consumption [35]. All these findings confirm the concept of moving away from the simple volumetric quantification of LA and extending the LA evaluation to a comprehensive analysis of LA function and LA dyssynchrony [36].

Moreover, the presence of LA enlargement among patients with LV hypertrophy appears to be correlated with the severity of the myocardial perfusion study defect via single-photon emission computed tomography (SPECT) [37]. Coronary microvascular dysfunction (MVD) may contribute to the pathogenesis of HFpEF; the assessment of coronary flow reserve (CFR) with positron emission tomography (PET) has a pivotal role in the diagnosis of MVD, aids risk stratification, and may be useful in monitoring therapy [38]. In patients with risk factors for HFpEF, MVD is associated with LVDD, increased estimated LV filling pressure, and abnormal LA strain [39].

### 3.2. Left Atrium in Heart Failure with Reduced Ejection Fraction (HFrEF)

LA enlargement and dysfunction are frequent phenomena in patients with HFrEF with various degrees of clinical implications, from AF occurrence to worse outcomes. LA abnormalities correlate with the increased production of renin/angiotensin/aldosterone, sympathetic stimulation, and markers of systemic inflammation (i.e., C-reactive protein), which are significant pathways of HF. In patients with HFrEF, due to nonischaemic dilated cardiomyopathy, the E/E’ ratio, left atrial dimension, and NT-ProBNP predict cardiac events and give incremental prognostic values beyond conventional risk factors [40]. This is confirmed in a large meta-analysis of 1157 patients: The LA area is a powerful predictor of outcome among HF patients with predominantly impaired systolic function. It is an independent factor with prognostic discrimination independently from the LV systolic and diastolic functions [41]. In patients with severe systolic dysfunction (FE ≤ 35%), cardiac resynchronisation therapy (CRT) shows a beneficial reverse remodelling of LA size [42]. Moreover, Sahebjam M et al. found a relation between LA size and function with LV dyssynchrony in patients with HF; CRT could have an independent role to improve the LA function in responders [43]. The LAVI also allows the identification of the best candidates for CRT [44]. In chronic heart failure (CHF) patients, an impaired LA reservoir function assessed through LA reservoir strain is linked with a severely reduced mixed venous oxygen tension (pVO_2_) independently from LVEF and known factors of reduced exercise capacity [45] (Table 1).

## 4. LA Size and Function in Coronary Artery Disease

LA activity is usually underestimated in patients with CAD, but recently, the evaluation of LA dimension and performance are studied in the pathophysiology of the ischaemic heart. LA is perfused primarily by the left circumflex coronary artery (LCx). Patients with proximal LCx stenosis develop ischaemia of LA branches, with an impaired LA performance that is unable to compensate for LV supply or demand during ischaemia [46]. Pinar et al. compared the left and right atrial size and function in acute coronary syndrome (ACS) patients with healthy controls, using transthoracic echocardiography by an evaluation of diameters, areas, volumes, and TDIs. They found higher LA and right atrial (RA) dimensions in ACS patients, compared with healthy controls. Similarly, the empty fraction and expansion index values of LA and RA were lower in ACS patients than in controls [47]. After ACS, the LA and LV are both affected, and cardiac remodelling is strongly associated with an increased risk of adverse CV outcomes. In daily practice, the evaluation of LA, in terms of morphology and function and risk stratification after an ischaemic event, is not adequately considered. However, many studies show that the LAVI after discharge of acute myocardial infarction is an independent incremental risk of HF rehospitalisation, all-cause mortality (ACM), and major adverse cardiovascular events (MACEs) [48]. Moreover, LA remodelling is common after anterior MI and correlates with the size of the scar. In the same way, LA electromechanical function is impaired in acute inferior STEMI and improves after revascularisation. The partial recovery could be due to either the resolution of the ischaemic disease or a response to an impaired LV diastolic function. The residual LA electric and contractile dysfunction hints at intrinsic pathology, likely to be ischaemic in origin [49]. Advanced techniques comprising the analysis of LA strain provide additional prognostic value beyond LA volume in terms of ACM, reinfarction, and hospitalisation for HF in patients with ACS treated with revascularisation therapy [50]. LA strain values decrease consensually to LV function in acute MI [51]. Specifically, global LA strain is lower in acute myocardial infarction (AMI) patients with a culprit lesion in the LCX than those with culprit lesions in other vessels, not associated with a significant difference in the LAVI. The impaired LA strain seems to be related to the impaired LA performance due to ischaemic insult. Moreover, changes in the LA fractional area and LA ejection fraction assessed by using computed tomography (CT) are more accurate than the LAVI to predict mortality in patients with non-ST-elevation myocardial infarction (NSTEMI) [52]. This is also confirmed by CMR images, where LA fractional change and volume are independently associated with outcome in ST-elevation myocardial infarction (STEMI) [53] (Table 2). Moreover, severe left atrial enlargement in patients with end-stage renal disease is independently associated with the presence of silent myocardial ischaemia assessed via SPECT. LA dilatation could be a potential marker for identifying patients with advanced renal disease at high risk of silent myocardial ischaemia [54].

## 5. Left Atrial Size and Function in Mitral Regurgitation

Functional mitral regurgitation (MR) is a consequence of mitral annular enlargement, leaflet tethering, and reduced coaptation. MR is an important cause of the modification of LA shape and function. LA dilatation leads to an enlargement of the mitral annular area and reduced leaflet coaptation even without LV dilatation. A coaptation index using three-dimensional transoesophageal echocardiography to evaluate MR provides insights into the mechanism of “atrial mitral regurgitation” [55]. In functional MR, an effective regurgitant orifice area (EROA) ≥ 0.30 cm^2^ is associated with CV events regardless of the LA function. Nevertheless, in EROA ≥ 0.10 cm^2^, a reduced LA function (PALS < 14%) is related to a worse outcome, providing measurable proof of the interplay between regurgitation and LA compliance [56].

In chronic MR, the LA changes its anatomy and becomes more spherical with a decrease in the LA eccentricity index according to the MR severity. This anatomical change is the expression of volume overload secondary to MR. The LA eccentricity index, an indicator of changes in the structure of the LA, is related to LA systolic function in chronic MR [57]. The continuum of the remodelling process starts in the early phases when mitral regurgitation is only mild/moderate. In this clinical setting, the left atrium, through pathophysiological adaptation, is able to maintain a normal SV, until a maladaptive remodelling prevails. This is demonstrated in a recent paper in which in a mild degree of MR, EROA was significantly associated with worse measures of LA volume and strain, also after adjusting for clinical and echocardiographic confounders [58]. The process of LA remodelling is strongly correlated with a loss of compliance and stiffness that could be detectable in clinical practice with myocardial deformation analysis even before LA enlargement and dysfunction are evident.

The presence of CV risk factors such as diabetes mellitus may further aggravate LA remodelling, leading to impaired LA deformation. Age and diabetes mellitus are linked to a decrement of LA strain [59]. Moreover, in patients with CV risk factors and evidence of ischaemic heart disease, the myocardial perfusion pattern in the left ventricular segments adjacent to the papillary muscles influences the presence and severity of MR that is significantly related to the left atrial enlargement and dysfunction [60]. Recently, the European Society of Cardiology Guidelines for the management of valvular heart disease suggested surgical mitral valve repair in low-risk asymptomatic patients with LVEF > 60%, LVESD < 40 mm, and significant LA dilatation (volume index > 60 mL/m^2^ or diameter > 55 mm), highlighting LA remodelling as central in MR surgery [61]. Mitral valve surgery (MVS) for chronic degenerative MR leads to LA reverse remodelling characterised by LA volume reduction and an improvement of LA function. LA size reduction after surgery is a potential marker for postoperative reverse remodelling and surgery success. However, the authors considered the absence of postoperative LA reverse remodelling not associated with an increase in postoperative mortality or adverse clinical events [62]. Instead, Debonnaire et al. revealed that impaired LA reservoir strain in patients with severe organic MR is linked to a worsening survival rate after mitral valve surgery [63]. In patients with chronic severe MR who received successful MV repair surgery, preoperative LA GLS is independently associated with long-term postoperative outcomes [64]. In a large international multicentre study, an LA diameter of ≥55 mm is associated with mortality and worse outcomes, independently of HF symptoms or LV dysfunction in patients with MR caused by flail leaflets. LA enlargement is a prognostic indicator in this type of MR and should be analysed in the context of routine clinical practice [65]. This assessment could be useful in the currently used algorithm for risk stratification and decision making [66] (Table 3).

## 6. LA Size and Function to Assess Stroke Risk and before Ablation Procedure in AF

LA atrial remodelling is responsible for the initiation and maintenance of AF. Chronically elevated LA pressure leads to structural changes; the progression of LA remodelling is an independent predictor of AF incidence [67]. The complementary approach by the evaluation of LA volume, Doppler transmitral flow velocity, and TDI has a higher predictive value to identify patients with paroxysmal AF and stroke risk [68]. TDI A’ reflects LA pump function and the severity of LA remodelling with a significant association with LA blood stasis in patients with nonvalvular PAF [69]. The impaired LA reservoir and booster pump function represent independent risk factors to develop AF beyond LA volume and dimensions [70]. In permanent AF, speckle tracking echocardiography, with the evaluation of myocardial deformation by a decrease in peak positive longitudinal strain during atrial filling and peak strain rate in the reservoir phase, recognizes patients with higher stroke risks [71]. Electroanatomical remodelling of LA, estimated by LA volume (3D computed tomography images) and endocardial voltage (3D electroanatomical map), is related to the CHADS₂/CHA₂DS₂VASc score in patients with nonvalvular AF [72]. The low left atrial appendage flow velocity (LAAFV) in patients with AF is a marker of a higher risk of stroke events. The presence of functional abnormalities of LAA identifies patients predisposed to thrombus formation in the LAA and at higher risk for subsequent cardioembolic stroke. LAA function in clinical routine practice is assessed by LAA emptying velocities using transoesophageal echocardiography (TEE). Researchers have suggested measuring the emptying velocity near the LAA orifice instead of the LAA apex [73]. Recently, CMR has proven to be more accurate and precise to assess the anatomy and flow velocity of LAA [74]. LAA orifice dilatation could be the cause of decreased flow velocity in LAA and could be highlighted in patients with higher stroke risks [75]. The LAA emptying and filling are influenced also by LV function. This could explain the higher incidence of AF-related stroke in patients with LV dysfunction in comparison with patients with normal LV systolic function [76]. However, stable sinus rhythm after catheter ablation performed for persistent AF does not lead to LAAFV recovery. Further studies are mandatory to confirm the usefulness of LAAFV to predict thromboembolism events in patients with sinus rhythm after AF ablation [77].

### Left Atrium Evaluation before Ablation Procedure in AF

Pump et al. affirmed that catheter ablation in patients with nonparoxysmal AF is effective independently from LA enlargement and is associated with LA reverse remodelling and improvement in LV ejection fraction [78]. Moreover, other authors remarked that LA dimensions should not discourage surgeons when evaluating candidates for surgical ablation [79]. CMR LGE recently has proven to be a strong tool to detect fibrosis in the atria so as to identify areas susceptible to catheter ablation [80]. CMR LGE provides a noninvasive means of assessing LA myocardial tissue in patients with AF. Preablation CMR LGE predicts responders to AF ablation [24]. CMR LGE could have a significant role also during the postablation phase. LA wall thickening and oedema following pulmonary vein ablation correlate with a 30-day LGE scar [81]. Atrial regions ablated show a higher contrast uptake and thinner myocardium, compared with regions with pre-existing LGE. The association of postablation LGE characteristics such as intensity and uniformity are related to ablation success and could give incremental information about ablation success [82]. Fibrotic tissue in the atria can also be identified with high-voltage endocardial mapping via catheter investigation. Moreover, pre-existing LVAs, reflecting endocardial scar and atrial tissue with different degrees of structural defect and remodelling, are independent predictors of AF ablation failure. Targeting LVAs in addition to pulmonary vein ablation while performing AF catheter ablation has shown to be significantly more effective than both pulmonary vein ablation alone and pulmonary vein ablation + conventional wide empirical ablation, without increasing the rate of adverse events [83].

**Figure 3:**
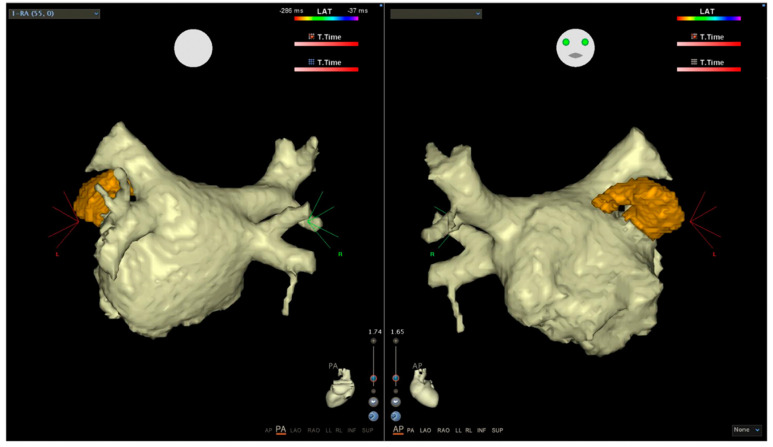
Reconstruction of the LA, pulmonary veins, and left appendage with the true anatomy using multislice computed tomography.

LA function determined by using 3D echo and clinical data (age, hypertension) predicts the recurrence of AF after the first ablation procedure, independently of LA size. Among patients undergoing repeated procedures, only a younger age and 3D echocardiographic LA volume are linked with no recurrence of AF after RFCA [84]. Many studies demonstrated that a dilated LA is a predictor of the failure of radiofrequency catheter or surgical ablation. Specifically, patients with LA dilatation, dysfunction, and fibrosis are more likely to experience AF recurrences after catheter ablation. The assessment of LA fibrosis via a two-dimensional echocardiography-derived calibrated integrated backscatter as a surrogate of LA fibrosis could be a predictor of new episodes of AF after radiofrequency catheter ablation (RFCA). A combined assessment of LA fibrosis and LA dimension could better identify patients with higher percentages of success after ablation procedures. LA myocardial deformation imaging, obtained by the measurement of LA size and function using longitudinal strain and strain rate during ventricular systole, is a reliable tool to predict success after a first and second RFCA. AF ablation requires a precise reconstruction of the LA and pulmonary veins (PVs) (Figure 3). Cardiac computed tomography (CT) can provide a precise tridimensional anatomic roadmap and exclude intra-cardiac thrombus. Recently, a model-based FAM (m-FAM) has been developed for the CARTO system which applies machine learning techniques with a precise reconstruction of the LA [85] (Table 4).

## 7. Conclusions

The left atrium is one of the leading actors of cardiac performance and cardiac neuroendocrine activity. New insights demonstrate significant associations between the LA structure and function measures with incident cardiovascular outcomes with potential clinical utility for the disease discrimination, outcome prediction, and management of patients with HF, CAD, AF, and valvular diseases. LA dysfunction represents a marker of poor prognosis across LVEF borders in the HF population; the LAVI and LA dysfunction significantly correlate with worse clinical symptoms and are red alerts of poor prognosis across HF phenotypes. The assessment of LA size, shape, and function, comprising different imaging techniques, could identify patients with worse atrial rearrangement and more advanced diastolic dysfunction. This should also be applied to patients with a higher supraventricular burden: A greater AF risk is associated with LA remodelling and more pronounced LA stiffness. The LA needs to be deeply investigated with a multimodality imaging approach, and precise cutoffs of LA size and function need to be better established through larger studies.

## Figures and Tables

**Table 1:** Instrumental parameters to evaluate left atrial function in HFpEF and in HFrEF and their clinical usefulness.

Clinical Setting	Parameter	Clinical Usefulness
HFpEF	E/E’	A noninvasive measure of LV filling pressure. Diagnostic and prognostic utilities to assess diastolic dysfunction.
	LAVI	Increased LAVI is an expression of increased LA pressure. It is a predictor of mortality in patients with HFpEF [31].
	Mean atrial pressure	It is a measure of LA compliance, which seems to be the most powerful independent predictor of limited exercise capacity [31].
	LAKE	It is a strong predictor of cardiovascular events and hospitalisation for HF.
	Atrial diastolic wall strain index	It is associated with worse outcomes in patients with HFpEF [35].
	Left atrial longitudinal strain (CMR)	It identifies subjects with diastolic dysfunction [36].
	LA conduit strain (CMR)	The conduit function is not significantly related to LV stiffness and relaxation, arguing that the LA conduit function reflects intrinsic LA pathology [37].
HFrEF	E/E’	A noninvasive measure of LV filling pressure. Diagnostic and prognostic utilities to assess diastolic dysfunction.
	LA area	LA area shows prognostic discrimination independently from LV systolic and diastolic functions [40].
	LAVI	Patients with low maximal LAVI are better responders to CRT than patients with higher values. It also allows the selection of candidates for CRT, being an independent predictor of LV reverse remodelling [43].

BSA: body surface area; CMR: cardiac magnetic resonance; CRT: cardiac resynchronisation therapy; E/E’: ratio between early mitral inflow velocity and mitral annular early diastolic velocity; HF: heart failure; HFpEF: heart failure with preserved ejection fraction; HFrEF: heart failure with reduced ejection fraction; LA: left atrial; LAKE: left atrial kinetic energy; LAV: left atrial volume; LAVI: left atrial volume index; LV: left ventricular.

**Table 2:** Instrumental parameters to evaluate left atrial function in coronary artery disease (CAD) and their clinical usefulness.

Clinical Setting	Parameter	Clinical Usefulness
CAD	LAVI	LAVI after AMI is an independent incremental risk factor for HF hospitalisation, ACM, and major CV events [31,47].
	LA strain	LA strain provides prognostic value in terms of ACM, reinfarction, and HF hospitalisation in patients with ACS treated with primary PCI [49]. LA strain values decrease consensually with LV function in AMI patients treated with revascularisation therapy, especially with culprit lesions in the LCx [50].
	LA FAC e LA EF (CCT)	LA FAC e LA EF evaluated by using CCT is more accurate than the LAVI to predict mortality in patients with NSTEMI [51]. This is also confirmed by CMR images, where LA FAC and volume are independently associated with outcomes in STEMI [52].

ACM: all-cause mortality; ACS: acute coronary syndrome; AMI: acute myocardial infarction; CAD: coronary artery disease; CCT: cardiac computed tomography; CMR: cardiac magnetic resonance CV: cardiovascular; EF: ejection fraction; FAC: fractional area change; HF: heart failure; HFH: hospitalisation for heart failure; LA: left atrial; LAVI: left atrial volume index; LCx: left coronary artery; NSTEMI: Non-ST-elevation myocardial infarction; PCI: percutaneous coronary intervention; STEMI: ST-elevation myocardial infarction.

**Table 3:** Instrumental parameters to evaluate left atrial function in the setting of MR and their clinical usefulness.

Clinical Setting	Parameter	Clinical Usefulness
Mitral Regurgitation	EROA and LA strain	In functional MR, EROA ≥ 0.30 cm^2^ is associated with CV events regardless of LA function. Nevertheless, in EROA ≥ 0.10 cm^2^, a reduced LA function (PALS < 14%) is related to a worse outcome [54].
	LA eccentricity index	LA eccentricity index is an indicator of changes in the structure of LA towards a more spheric geometry and is related to LA systolic function in chronic MR.
	LA anteroposterior diameter	LA enlargement is associated with mortality and worse outcomes, independently of HF symptoms or LV dysfunction in patients with MR caused by flail leaflets [60,61].

CV: cardiovascular; EROA: effective regurgitant orifice area; HF: heart failure; LA: left atrium/left atrial; LV: left ventricular; MR: mitral regurgitation; PALS: peak left atrial longitudinal strain.

**Table 4:** Instrumental parameters to evaluate left atrial function before AF catheter ablation and their clinical usefulness.

Clinical Setting	Parameter	Clinical Usefulness
Before FA catheter ablation	TDI	TDI A’ reflects LA pump function and the severity of LA remodelling with a significant association with LA blood stasis in patients with nonvalvular PAF [63].
	STE	The evaluation of myocardial deformation by a decrease in peak positive longitudinal strain during atrial filling and peak strain rate in the reservoir phase recognises patients with higher stroke risks [65].
	LA volume + Fibrosis evaluation (Eco)	Low 3D echocardiographic LA volumes are linked with no recurrence of AF after RFCA [77]. Patients with LA dilatation, dysfunction, and fibrosis are more likely to experience AF recurrences after RFCA.
	Left atrial appendage flow velocity (LAAFV)	Low LAAFV is related to a higher risk of stroke events.
	LA anatomy	CT reconstruction of LA anatomy is useful before a catheter ablation procedure.
	CMR LGE in the LA	Preablation CMR LGE predicts responders to AF ablation and may provide insights into the overall disease progression [23,24].
	LA volume (3D CT) + 3D electroanatomical map	This is related to the CHA₂DS₂VASc score in patients with nonvalvular AF [66].

AF: atrial fibrillation; CMR: cardiac magnetic resonance; LA: left atrium/left atrial; LAAFV: left atrial appendage flow velocity; LGE: late gadolinium enhancement; PAF: paroxysmal atrial fibrillation; RFCA: radiofrequency catheter ablation; STE: speckle tracking echocardiography; TDI: tissue Doppler imaging; 3D: three dimensional; 3D CT: three-dimensional computed tomography.

## Data Availability

All articles’ references can be found on the online platform Pub-med.gov and have been last accessed on 3 August 2022.
